# Feasibility of a Reablement Programme in Community Care in the Netherlands; A Qualitative Study

**DOI:** 10.5334/ijic.9005

**Published:** 2025-09-10

**Authors:** Lise Elisabeth Buma, Stan Vluggen, Ines Mouchaers, Sandra M.G. Zwakhalen, Silke F. Metzelthin

**Affiliations:** 1 Care and Public Health Research Institute, Department of Health Services Research, Maastricht University, Maastricht, The Netherlands; 2 Living Lab in Ageing and Long-Term Care, Maastricht, the Netherlands Cicero Zorggroep, Brunssum, The Netherlands; 3 Living Lab in Ageing and Long-Term Care, Maastricht, The Netherlands; 4 Academy of Nursing, Zuyd University of Applied Sciences, Heerlen, The Netherlands; 5 Department of Public Health and Primary Care, Academic Centre for General Practice, KU Leuven, Leuven, Belgium

**Keywords:** restorative care, interdisciplinary collaboration, behaviour change, patient-centred care, independence

## Abstract

**Introduction:**

Many older individuals face complex health and social care issues that require an integrated care approach. However, current health and social care services are often fragmented in terms of financing, organisation, and delivery, and therefore do not fit the needs of older individuals. Reablement is an innovative integrated approach that aims to ‘help individuals to help themselves’ by providing goal-oriented, person-centred care. An interdisciplinary team works intensively with individuals towards their goals, while considering their capabilities and contextual factors.

**Description:**

The aim of this study was to assess the feasibility of a Dutch reablement programme in terms of acceptability, implementation, practicality, adaptation, integration, and limited efficacy. Using qualitative methods, six clients, three informal caregivers, eight care professionals, and one programme director were interviewed, complemented by data from electronic care files. Findings indicated positive stakeholder experiences.

**Discussion:**

Despite positive experiences, three key challenges emerged: 1) behaviour change; 2) in- and external interprofessional collaboration; and 3) enrolment. The study underscores the complexity of implementing and integrating reablement in community care.

**Conclusion:**

Our findings offer critical insights that could guide future efforts to implement reablement programmes more effectively.

## Introduction

Most older individuals prefer to stay at home for as long as possible, as resembled by a national aging in place policy that focuses on providing support and services to older individuals to remain living in their familiar home [1]. Older individuals often have complex health and social needs that require a coordinated, holistic approach [2]. However, current care and social services are often fragmented in terms of financing, organisation, and delivery, hindering collaboration and coordination [3,4,5]. The movement towards a more integrated approach is necessary to avoid the unilateral focus of different professionals from their field of expertise and the consequent inability to coordinate care to the person’s needs [6,7]. Consequently, many countries show increasing interest in integrated care approaches. Integrated care requires a shift in the current healthcare system from merely focusing on care and wellbeing, towards providing demand-oriented care, focusing on the needs and preferences of individuals [8]. Moreover, care professionals must support clients to become more self-reliant by not taking over care tasks as has been done for many years [9].

Reablement is such an integrated approach that prioritises both care and wellbeing while promoting demand-oriented care [10]. Reablement is suitable for everyone regardless of age, diagnosis and setting [10]. Reablement aims to ‘help individuals to help themselves’ by delivering goal-oriented, person-centred care. An interdisciplinary team works intensively with individuals towards their goals, while considering their capabilities and contextual factors. The international movement towards reablement began to take serious shape from 2000 onwards when care organisations in various countries adopted reablement as an alternative to traditional home care services [11]. Currently, more than 15 countries have been or are currently in the process of integrating a reablement approach into their healthcare systems [12]. There are indications that reablement improves health and well-being, reduces care needs, and saves costs [13,14,15]. It also seems promising as it offers high satisfaction levels across all stakeholders involved including caregivers and care receivers [15,16,17]. However, the existing evidence about the effectiveness of reablement is inconclusive [18,19,20,21]. Next to methodological issues (e.g. variation in study designs, outcome measures) this may be explained by differences in the core components and characteristics of reablement programmes [21].

To address these variations and enhance the consistency and effectiveness of future reablement practices, recently, a reablement model was developed in co-creation with care professionals, policymakers, client representatives, informal caregiver (representatives), and scientific experts: the I-MANAGE model [22]. The I-MANAGE model can guide the design and implementation of reablement programmes in Dutch community care and can be tailored to the context in which it is implemented and consists of six essential components namely, improving assessment and goal setting; stimulating self-management during meaningful daily activities; optimising the use of the physical environment; optimising the use of the social environment; improving interprofessional collaboration; and supporting the informal caregiver. It consists of five chronological phases: 1) initiation, 2) intake, 3) care plan, 4) care delivery, and 5) evaluation [22]. It emphasizes interdisciplinary collaboration and includes practice-oriented training. During eight to twelve weeks, the individual and their informal caregiver(s) are supported and monitored by a dedicated reablement team. Details regarding the specific content and development of the I-MANAGE model are described elsewhere [22].

The I-MANAGE model was used to guide the development of a reablement programme for community care. The aim of this study was to assess this programme’s feasibility in terms of acceptability, implementation, practicality, adaptation, integration, and limited efficacy following the feasibility framework of Bowen et al. (2009) [23], using a multi-stakeholder perspective.

## Methods

### Study design

A qualitative feasibility study was conducted between April 2022 and July 2023, assessing the programme’s feasibility according to the constructs of Bowen’s feasibility framework [23]. This framework was chosen due to the exploratory and small-scale nature, offering a pragmatic approach allowing for preliminary insight into potential effectiveness through limited efficacy testing. The data comprised individual interviews and a focus group interview to explore stakeholders’ experiences with the implemented reablement programme.

### Setting

The study was conducted at a long-term care organisation in the southern part of the Netherlands, providing various forms of clinical and long-term care. As part of the organisation’s strategy to sustain care services and empower older individuals to maintain their independence at home for as long as possible, the organisation has initiated a pilot project to implement reablement in community care using the reablement model I-MANAGE [22]. Several implementation strategies were applied during the pilot, including early stakeholder engagement, educational sessions on reablement, contextual adaptation, and ongoing coordination by a project lead. These reflect common strategies such as promoting adaptability, tailoring strategies, interactive assistance, and strengthening stakeholder relationships [24]. A separate study provides a more detailed account of the implementation experiences [25].

### Implementation of the reablement programme

The care organisation used and tailored the I-MANAGE model (i.e., team composition, target group) to implement reablement. A detailed description of the reablement programme can be found in Appendix 1. The interdisciplinary team consisted of an occupational therapist (OT), physical therapist (PT), informal care consultant, community care nurse (CCN), and an elderly care physician. Other care professionals were consulted based on the goals set by the individual. The OT conducted the initial assessment using Positive Health principles [26], with additional assessments by other disciplines if needed. Care professionals were trained in principles of reablement and Positive Health [26], and OTs received specific training on the coordinator role and Canadian Occupational Performance Measure (COPM) [27]. The COPM is a goal-setting tool which uses a semi-structured interview to capture a client’s self-perception of performance in areas of self-care, productivity and leisure in terms of satisfaction and performance [27]. PTs received a brief OTAGO training and CCNs were briefed about the programme during one of their team meetings. OTAGO is a tailored balance and strength fall prevention programme [28].

The programme targeted community-dwelling older individuals receiving community care with a decline in daily functioning, referred by the CCN, general practitioner or elderly care physician, excluding those receiving rehabilitation, with terminal illness, or severe cognitive impairment.

### Sampling and recruitment

Participants were individuals participating in the reablement programme, their informal caregivers, members of the reablement team and the programme director of the care organisation. The OTs asked all older individuals involved in the programme, alongside their informal caregivers to participate in the study. Criterion sampling was used by the project leader of the organisation to select professionals [29]. In addition, the programme director was also asked to participate. Eligible professionals had to be involved during the development, deployment, and/or implementation of reablement ensuring a well-rounded representation of professionals. All study participants were expected to be proficient in Dutch and provide informed consent.

### Data collection

Data were collected in line with the feasibility aspects from Bowen et al. (2009) [23]; implementation, adaptation, integration, acceptability, practicality, and limited efficacy testing. An overview of the research methods used can be found in Table 1.

**Table 1:** Data collection according to feasibility concepts of Bowen et al. (2009) [23].


AREA OF FOCUS	DEFINITION	DATA COLLECTION			

		DATA SOURCES	DATA COLLECTION METHODS	OPERATIONALISATION	TIMING OF DATA COLLECTION

Implementation	To what extent can a new idea, programme, process, or measure be successfully delivered to intended participants in some defined, but not fully controlled, context?	Care professionals Programme director Researchers	Focus group interviews Field notes Electronic care files	Facilitators and barriers of the programme’s implementation Perceived quality of implementation Adherence to protocol	After implementation Throughout implementation

Adaptation	To what extent does an existing idea, programme, process, or measure perform when changes are made for a new format or with a different population?	Care professionals Programme director Researchers	Focus group interviews Field notes Electronic care files	If applicable: changes in content, procedures, activities, and processes	After implementation Throughout implementation

Integration	To what extent can a new idea, programme, process, or measure be integrated within an existing system?	Care professionals Programme director Clients and informal caregivers Researchers	Focus group interviews Semi-structured interviews Field notes	Perceived fit with infrastructure Perceived sustainability	After implementation Throughout implementation

Acceptability	To what extent is a new idea, programme, process, or measure judged as suitable, satisfying, or attractive to programme deliverers? To programme recipients?	Care professionals Programme director Clients and informal caregivers	Focus group interviews Semi-structured interviews	Perceived satisfaction, appropriateness, and intent to continue use Perceived positive or negative effects on organisation Opinion about the programme	After implementation

Practicality	To what extent can an idea, programme, process, or measure be carried out with intended participants using existing means, resources, andcircumstances and without outside intervention?	Care professionals Programme director Clients and informal caregivers	Focus group interviews Semi-structured interviews Electronic care files	Engagement with the programme and application in practice Positive/negative effects on target participants	After implementation

Limited efficacy	Does the new idea, programme, process, or measure show promise of being successful with the intended population, even in a highly controlled setting?	Care professionals Programme director Clients and informal caregivers	Focus group interviews Semi-structured interviews Electronic care files	Intended pre- and post-effects of programme	After implementation After implementation


Background characteristics (e.g., age, sex) were collected from all participants. To reduce burden of data collection, electronic care files tracked client progress and goal related outcomes, including COPM goals, used interventions, progress reports, and final outcomes. Semi-structured interviews with clients and informal caregivers were conducted by researcher LB at participants’ residences after programme completion, focusing on goal setting, care received, informal caregiver roles, shared decision-making, points for improvement, and overall experiences. The interviews were conducted following a topic guide based on Bowen et al. (2009) [23].

Simultaneously, at one of the locations of the care organisation a focus group involving care professionals was conducted by researchers LB and SM, discussing interdisciplinary collaboration, care delivery, barriers, facilitators, points for improvement, and programme experiences. In addition, a semi-structured interview with a similar topic guide was conducted with the programme director by researchers LB, IM, and SM.

The interview data were supplemented by field notes taken by the researchers during and after the interviews to capture intricate details. The interviews were audio-recorded, anonymised, and transcribed verbatim using simple orthographic notation. Additional field notes were used to capture intricacies observed during the implementation and execution of the programme. Interview guides are available in Appendices 2–4.

### Data analysis

Content analysis [30] was conducted by researcher LB and reviewed by researchers SV or SM to extract information related to Bowen et al.’s areas of focus within the interview data [23]. The researchers familiarised themselves with the data by reading the transcripts several times while making notes. The transcripts were then imported to Atlas.ti software (Windows Version 23.4.0) for coding. Relevant segments were identified, coded and categorised following the areas of focus by Bowen et al. (2009) [23] by researcher LB and discussed with researchers SV and SM. Codes were refined and organised into a matrix by researchers LB and SV, mapping key areas across stakeholder groups (Appendix 5). This matrix allowed for comparison and synthesis of insights. The analysis was iterative, with ongoing discussions and arbitration to resolve disagreements between all researchers. Fieldnotes were compared with the data to assess implementation, adaptations, and programme integration.

Demographic characteristics of the participants were analysed using descriptive statistics. Frequencies and percentages were calculated for categorical variables (e.g. sex, educational level, employment status), while mean and standard deviation were computed for continuous variables (e.g. age). Data analyses were performed using SPSS version 27.0 (IBM Corp., 2020).

### Ethical considerations

The study was reviewed and approved by the FHML Research Ethics Committee of Maastricht University (FHML-REC/2022/048). Informed consent was provided, and all participants received information about the study’s purposes and the right to withdraw from the study. All data was anonymised and stored on the research server of Maastricht University.

## Results

Data was collected from a total of six clients participating in the reablement programme, three informal caregivers, eight care professionals and one programme director. The sample of care professionals consisted of two PTs, two OTs, one CCN, one elderly care physician, one informal care consultant, and one nurse practitioner. The background characteristics of all participants are displayed in Table 2.

**Table 2:** Background characteristics of participants (n = 18).


	CLIENTS AND INFORMAL CAREGIVERS(n = 9)	CARE PROFESSIONALS AND PROGRAMME DIRECTOR(n = 9)

Age (years), mean (SD)	78.4 (6.0)	45.2 (11.2)

Sex, n (%)		

Male	3 (33)	

Female	6 (67)	8 (100)

Educational level*, n (%)		

Intermediate		

High		9 (100)

Years of experience, mean (SD)		

Professional role		11.9 (7.7)

Reablement		2.0 (0.0)


Notes: * Intermediate: Intermediate vocational or higher secondary education; High: Higher vocational or university education.

All data collected (Table 1) related to five domains of feasibility as outlined by Bowen et al. (2009) [23]. The results were merged into three core sections; (1) Implementation, adaptation, and integration; (2) Acceptability and practicality; and (3) Limited efficacy. Within each section, the collected data are synthesized to offer a thorough depiction of the feasibility. Concluding the results section, a case description based on the collected data is included to illustrate the programme’s feasibility.

### Implementation, adaptation, and integration

According to the field notes, new clients were usually enrolled in the programme through referral of their general practitioner and/or CCN, through the elder care physician following their assessment, or following clinical rehabilitation. During the initiation phase, care professionals also conducted eligibility screenings to determine which clients qualified for the programme. However, due to insufficient client enrolment, eligibility criteria were broadened to the extent that severe cognitive problems, terminal illness, and lack of support from the client’s social network were the only exclusion criteria remaining. Within the focus group interview, care professionals expressed that a too strict eligibility screening may have been the main cause of insufficient client enrolment. In addition, they indicated occasional problems due to miscommunication in team meetings when discussing new referrals, possibly leading to uncertainty about a client’s eligibility to enter the programme.

During the focus group interview, OTs experienced that the exploratory conversation using the Positive Health questionnaire proved beneficial for setting goals during the intake assessment. They indicated that this helped them to better understand the client’s situation.

“So, I believe that because there is a lot of attention given to the intake of the reablement programme, it allows you to… I think by asking questions differently, or perhaps when the question is posed by someone else, you can get to the fundamental parts more effectively.” – Community care nurse

Initially, administering the Positive Health questionnaire and COPM involved two separate home visits. However, OTs found the separate visits approach time-consuming and opted to send the Positive Health questionnaire to the client by e-mail before the assessment.

To formulate the care plan, the first team meeting was typically scheduled within the initial two weeks of the programme. However, within the focus group interview, care professionals indicated that occasionally it was scheduled prematurely, resulting in rescheduling due to insufficient time to gather all necessary information. After the first enrolled client, the reablement team decided that the intake by the informal care consultant should be scheduled after the OTs initial assessment, as it provided them with more background information prior to their intake with the informal caregiver. Moreover, care professionals indicated it was often difficult to schedule team meetings due to the large team size. Over time, they felt the whole process became more efficient, but expressed the need for fixed timeslots for team meetings and clear guidelines and logistics regarding work processes. In addition, occasionally, some interventions or therapies were initiated later than planned as not all necessary care professionals were available initially. When asked about the implementation of coaching on the job during care delivery by the team, care professionals indicated this was implemented occasionally and rather unconsciously.

The care plan was documented in the electronic care file, but access to all reports and the reablement plan was limited to care professionals within the organisation. Consequently, individuals in the focus group expressed that external professionals were therefore reliant on the team meetings to follow progress and actions taken toward achieving clients’ goals.

In addition, care professionals indicated that within the electronic care file, it was often unclear who was already involved, and existing forms were not user-friendly and difficult to modify. Furthermore, it became evident that the reporting of the COPM was not carried out consistently, possibly because it was a new part of the procedure. As a result, pre- and/or post-assessment data were incomplete for four of the six clients. This inconsistency was also observed in other areas, as gaps in reporting were identified during the analysis of the electronic care files.

As documented in the electronic care files, coordination was organised by the OT, personal care needs by the CCN and functional training and stimulating participation by the PT. When requested, additional support for the informal caregiver was provided to decrease their burden. Overall, the five phases of the reablement programme, shaped according to the I-MANAGE model, were delivered as intended. However, the programme was extended twice: once due to the temporary unavailability of a psychologist, and again because the client needed more time to achieve their goals.

### Acceptability and practicality

In the interviews, clients indicated that their interaction and collaboration with the reablement team was pleasant and highly appreciated. They appreciated that the team looked beyond just their status as patients to find solutions together. Moreover, some clients mentioned that actively participating in the tasks and goals based on the exploratory conversation was satisfying. Clients felt listened to, connected on a personal level, involved during the process, and empowered to decide for themselves. Informal caregivers expressed high satisfaction with the programme and their interaction with the team, feeling supported and able to rely on them.

“Both of us were truly impressed by the dedication of all those involved; they truly make things happen, and we have greatly benefited from their efforts. We have nothing but praise in this regard. […] Overall, I feel they genuinely went above and beyond for us, which has been truly remarkable. While at times it felt like a lot, in general, their efforts were effective.” – Informal caregiver 2

In addition, clients perceived the team’s collaboration with already involved external care professionals as good, mentioning regular communication and exchange of information at the end of the programme as positive attributes. Generally, informal caregivers were positive about the guidance and practical support from the informal care consultant but mentioned repetitive questions from different care professionals.

During the focus group interview, care professionals expressed that the scheduled team meetings helped them to align and coordinate tasks within the team and to regularly assess care delivery concerning the established goals. In addition, OTs felt supported by the care organisation in organising the reablement programmes, as they were allocated specific hours for coordination. The importance of effective coordination among the different stakeholders involved was emphasised during interviews with care professionals and the programme director, especially due to overlapping responsibilities. Care professionals indicated that they experienced the various perspectives of each team member as valuable. The fact that the community care nurse and the informal care consultant were now structurally involved in the meetings through the programme were mentioned as a promoting factor. Positive Health [26] and interdisciplinary collaboration also facilitated different perspectives, leading to a broader and deeper understanding of the issues, which was positively perceived by both clients and care professionals.

“By administering the Positive Health questionnaire beforehand, you can already bring up a few more things that you can discuss. […] So usually they’ve already filled it out, and then I have more time to delve into it more deeply. So, then I already know roughly where the problems lie, and they’ve also been thinking about it a bit more. And then in the second meeting, when I administer the COPM, they can indicate their goals or request assistance more consciously.” – Occupational therapist 1

Care professionals highlighted the significance of having a fixed team to successfully deliver the reablement programme feeling certain experience and expertise was necessary. They indicated this became apparent when external care professionals were involved, as not sharing the same approach and mindset could hinder achieving clients’ goals. Additionally, care professionals felt the general practitioner’s involvement was insufficient, with a lack of feedback and communication, possibly because they were not sufficiently familiar with reablement.

“But I do think that it is very important that there is a fixed team of experts involved. People who know exactly what it’s (reablement) about and who have a better understanding of it, […] I think it also needs to suit you. It’s a different way of doing your job. And it’s not just a standard home visit. You have to be able to build a certain level of trust in a very short time. If you want to administer that (Positive Health questionnaire), people need to be able to show a certain vulnerability.” – Nurse practitioner

Moreover, the programme director mentioned that friction arose when external care professionals, such as a case manager, who typically fulfil a coordinating role within community care, were involved. This tension mainly stemmed from unclear role definitions within the programme, particularly in relation to other team members, with the programme director explaining that unclear role agreements create confusion and lead to feelings of friction.

The focus group revealed ambiguity about the programme’s content and purpose due to its confusing name and a lack of familiarity among community care nursing teams. The programme field notes showed that only CCNs had received information regarding the programme and were responsible for disseminating it to the rest of the team. In the interview, the programme director highlighted the importance of generating support from various stakeholders and the time needed for behaviour change.

“What you see is that we won’t achieve it with only an informative strategy. […] The seduction strategy of the behaviour change […] also […] underlies, right, to move from ‘doing for’ to ‘doing with’, you really must load (behaviour change) that as well. And […] that’s a multi-year process. That’s not something you accomplish in one year. And […] you still need to pay attention to […] behaviour change, and how you guide it in your organisation in a good way.” – Programme director

The programme director, care professionals, and clients frequently mentioned a change towards a reablement mindset because of the programme’s implementation. Moreover, care professionals were aware of the urgency for change due to current societal challenges, emphasizing its necessity for both clients and informal caregivers. However, they did regularly experience conflicts with clients and informal caregivers regarding their views on care delivery, negatively influencing the course of the programme. They indicated clients and informal caregivers often assert that they “have the right” to receive care, and still expect to receive care as promoted in the past by the government’s welfare state. However, this sentiment was not mentioned by the clients and informal caregivers themselves during the interviews.

“And that clashes regularly (different mindsets on care delivery). People also think […] that they have a lot of rights to things. Because they have worked their whole lives and they have paid a lot (health insurance), and now they deserve certain privileges. They often tell me, ‘You can simply come over because we’ve been paying for health insurance our entire lives, so we have every right to this; therefore, you must comply’.” – Community care nurse

OTs indicated that prioritising the goals with the client often led towards more practical and achievable goals. They mentioned it was crucial that clients formulated their own goals for intrinsic motivation and successful outcomes. This was confirmed by clients, stating that a goal-oriented approach motivated them. Clients emphasised that incorporating their social and physical environment promoted their recovery. Informal caregivers experienced significant support from the team but considered a good connection to be essential. Most clients indicated the care delivered was person-centred and each step taken was clearly explained, although some had trouble recalling programme details. Additionally, informal caregivers felt the delivered interventions were well aligned with the clients’ goals. They indicated the team consciously articulated the set goals during care and therapy sessions to emphasise the importance of certain interventions or activities. However, most clients struggled to recall these goals and felt they were not reiterated during the programme.

Supporting materials and technology promoted the implementation according to care professionals, for example, the use of videoconferencing for the team meetings increased flexible scheduling. Further mentioned facilitators were the time-limitedness of the programme and the team working on a common set of goals. The programme director identified funding as a clear barrier for the programme, as existing reimbursements were often too restrictive for the delivered care and support. Care professionals acknowledged this, indicating the care and support they deemed necessary could not always be delivered due to reimbursement restrictions.

Other barriers during implementation in terms of time were experienced, as the execution of the programme asked for significant time investment from the coordinator and the 8-week duration caused problems in the delivery of supporting services. For example, municipality waitlists for supporting services often exceeded the 8-week duration. In addition, there were some doubts whether the 8-week period was sufficient to establish behaviour change. Care professionals also mentioned the higher burden of the programme on both clients and their informal caregivers due to its short but intensive character, especially during the first two phases of the programme. This sentiment was shared by a client and informal caregiver as well.

“What I do feel is […] it was a lot to handle, especially at a time when (client’s name) experienced a significant setback, making things much worse. […] With all those limitations, I found it to be quite overwhelming.” – Informal caregiver 2

### Limited efficacy

Overall, both interview data and information from electronic care files indicated that most clients progressed in their functioning and were able to engage more in activities important to them. Examples are, being able to shower (again), cook, build and maintain social connections, and being able to walk to the community centre. One client initially hesitated to participate in day care activities but became convinced of their value after trying them once, reflecting a mindset shift resulting from the programme. An informal caregiver noted that the advice provided by the team was often more readily accepted by the client, facilitating a smoother transition towards change. Two clients faced setbacks during their programme due to unforeseen circumstances but felt supported by the team in overcoming these setbacks.

Interview data showed that informal caregivers were very satisfied with the progress achieved. They experienced more freedom to engage in personal activities, became more comfortable relinquishing tasks, learned to approach things differently, felt relieved from their concerns and were able to express their thoughts and worries openly with the team. The support of the team empowered informal caregivers and made them feel confident to look for solutions together with the client. Informal caregivers felt supported by the team and capable of looking for solutions together with the client. When asked whether the programme had impacted their relationship, they reported no differences.

“(When my partner is at day care) I can go shopping or take the dog for a long walk. […] because you’re just worried: what will he do? Will I find him injured again? He fell last Friday; he fell again on Monday.” – Informal caregiver 1

In the focus group, care professionals expressed high satisfaction with the programme’s results, mentioning improved communication and insight into each other’s professions due to the more intensive collaboration. However, they noted that maintaining client’s progress over time would require significant attention as the programme currently does not include a structural form of aftercare. In addition, the programme director emphasised the challenge of assessing the programme’s impact, highlighting the importance of long-term follow-up to evaluate its effects.

“In the post-care phase, I noticed that we need to make some improvements. […] I remained involved with three reablement clients after they completed the programme. And I do notice that ensuring sustained actions is still a challenge. One client […] reverted to old habits just a month later. This happened because she lost her motivation, and at that moment, the caregiver also didn’t know how to guide her, purely because we were no longer involved. He didn’t have that support anymore.” – Occupational therapist 2

### Exemplary case

**Box 1 Case description of a client and their informal caregiver within the reablement programme**

**Emily’s situation**

Emily (70) has an autoimmune disease that severely limits her mobility. She spends most of her time in a chair downstairs. “I hardly get out of the house anymore, even moving around inside is a challenge.” Her husband, John (71) acts as her primary caregiver. Home care supports with bathing twice a week, John manages the rest. Due to delirium, Emily’s care became too demanding, prompting a referral for reablement

**Goal setting**

Together with the OT, Emily determined what was most important to her. She set using the COPM:

1. Walking with a mobility aid i.e., to attend medical appointments.
2. Climbing stairs to shower independently upstairs, except for washing her lower legs and feet.
3. Participating in household tasks, including dusting and preparing meals with her husband.

John also set personal goals the informal care consultant, focusing on letting go of responsibilities and seeking support with household administration, a task he had taken on due to Emily’s condition. “Because Emily has been ill for years, I tend to take over more and more tasks.”

**The reablement programme**

Over eight weeks, the reablement team – comprising an OT, PT, psychologist, informal care consultant, and elderly care physician – worked with Emily and John on their goals. Biweekly team meetings tracked progress and adjusted the programme as needed. The OT assessed their home and helped Emily to explore ways to actively participate in the household again. The PT focused on walking and stair climbing, enabling her to shower upstairs. Emily also did balance and strength exercises. The psychologist addressed delirium-related needs, while the informal care consultant assisted John with administration and emotional well-being.

**Emily’s and John’s achievements**

After eight weeks, Emily was pleased: “Not everything worked out, but many things did.” She now showers upstairs with assistance, cooks with her husband, and dusts independently – to Johns relief, as he dislikes it. Though she still sleeps downstairs due to bathroom trips and discomfort with climbing stairs at night, she improved both performance and satisfaction scores on her COPM goals. John feels more able to leave the house briefly and taking some time for himself, though not for extended periods, as he is still adjusting to these changes.

## Discussion

The aim of this study was to assess the feasibility of a reablement programme based on the I-MANAGE model [22] in the community care setting. The study employed a qualitative design to assess the programme’s feasibility in terms of acceptability, implementation, practicality, adaptation, integration, and limited efficacy following Bowen et al. (2009) [23]. The results of our study reflect positive experiences, implementation challenges, fragmentation in healthcare, coordination issues with external professionals, and the need for cultural shifts towards a reablement mindset. In our discussion, we would like to elaborate on three challenges that stood out most clearly across themes to enable further reflection on the conditions that influenced feasibility. The three key challenges were: 1) behaviour change; 2) interprofessional collaboration both within and outside the organisation; and 3) enrolment.

The first challenge concerns behaviour changes towards a reablement mindset among all internal and external professionals involved. Our results show that professionals seem to struggle with inconsistent approaches and mindsets, and behavioural changes were not always sustained. This aligns with international research, which emphasise the need for structured support to sustain behavioural change for professionals as well as clients and informal caregivers involved [31]. Similarly, Beresford (2019) [32] found that without sustained support, professionals reverted to traditional care models, highlighting the need for comprehensive training and organisational support. Additionally, behaviour change requires time, communication strategies, collaboration mechanisms, and integrated training [33]. This requires structural support, such as team meetings and time to promote self-reliance [34].

The second challenge is interprofessional collaboration, especially with external care professionals. For example, current reablement training is centred on the interdisciplinary team within the care organisation potentially leading towards a compartmentalised approach, hindering the delivery of integrated care and full integration of the programme in the community beyond the care organisation itself [25,35,36]. International experiences highlight the necessity of alignment among all stakeholders [32,37]. Moreover, reimbursement structures in Scandinavian are structured to incentivise collaboration rather than competition among care providers [38]. A supportive system is needed that enables this approach, rather than allowing reimbursement structures to dictate the care provided as indicated by the care professionals in our study [39]. The extent to which the organisation was ready for reablement, and how structures enabled or hindered collaboration, directly influence the feasibility of implementation.

Third, a too strict selection process at the start may have resulted in a low programme enrolment rate and the inclusion of participants with substantial and higher care needs only. The strict selection process and the approach of those conducting screening may have affected enrolment [40]. Furthermore, connecting to the topic of eligibility, participants were often referred by general practitioners, reflecting a more reactive approach [41]. Adopting a more preventative approach could be beneficial in identifying individuals at risk before they require more expensive care [41]. From a feasibility perspective, this suggests that current screening procedures may hinder practical implementation and that improving them could enhance the accessibility of the programme to a broader range of suitable clients.

Studies on the feasibility of reablement programmes across different countries support our findings, showing that while reablement is promising, its success depends on contextual adaptation and adequate support for clients and caregivers [31]. Common challenges include coordination, training, interprofessional collaboration, and healthcare fragmentation [32,42,43,44]. Studies also stress the importance of user involvement and personalised care, reinforcing the value of co-creation in reablement programmes [45]. Moreover, while our findings align with international experiences, the implications for the Netherlands need further exploration [25]. The Dutch system—characterised by fragmented funding streams and a strong distinction between care domains—presents unique barriers and opportunities for scaling up I-MANAGE. For instance, current financing structures may complicate collaboration across organisational boundaries. At the same time, the growing focus on prevention and self-reliance within national policy could facilitate wider acceptance [46]. Future implementation efforts should therefore consider region-specific structures, local readiness, and inter-organisational dynamics. Developing tailored implementation strategies per region or organisation may enhance scalability across the Netherlands [47].

Our study has some limitations that need to be acknowledged. For instance, our results are based on a small study sample and there may be other perspectives not captured in the findings. Moreover, while Bowen’s framework aligned with our research aims, alternative frameworks might have highlighted additional aspects such as mechanisms of change, adoption, or long-term sustainability [23]. Additionally, care professionals’ limited exposure to clients may reduce generalisability and although implementation strategies were embedded in the process, their effectiveness was not formally assessed. However, a strength in our study is providing detailed insights into the experiences and perspectives of various stakeholders. This helped to highlight important factors, that may not be evident through quantitative methods, which are useful to tackle challenges during future implementation of reablement programmes. It is important to note that, involvement of older adults and informal caregivers in this study aligns with the consultation level of the ladder of participation [48]. Participants were actively consulted to share their experiences, provide feedback, and highlight areas of importance related to the reablement programme. Importantly, lived experience informed the development of the I-MANAGE model used in this study (22). The works and patient councils were involved in decision-making, ensuring organisational support. Lastly, several implementation strategies were employed that implicitly addressed contextual factors. While not central to our aims, a formal analysis could have offered valuable insights into factors affecting implementation. We recommend future research incorporate contextual analysis to help interpretation of feasibility findings and guide tailored implementation, using contextual determinants as reviewed by Nilsen and Bernhardsson (2019) [49] providing useful direction.

## Lessons learned

Despite its small-scale and limitations, our study can suggest several courses of action for policy and practice to promote further integration and ensure the feasibility of reablement programmes.

- Management and organisational policies must facilitate sustained behaviour change through structural processes to avoid reverting to old routines.
- Address siloed care by promoting cross-organisational collaboration, interdisciplinary training, and policies aligned with reablement principles, including reimbursement models.
- Expand client inclusion criteria to adopt a preventative approach, enabling early interventions, enhancing reablement’s effectiveness, and ensuring timely support.

## Conclusion

Overall, the reablement programme implemented in Dutch community care resulted in positive stakeholder experiences. At the same time, it highlights the complexity of implementing and integrating reablement in community care, thereby revealing three key challenges including behaviour change, interprofessional collaboration, and client enrolment. As our results closely align to results from various international reablement feasibility studies, our findings add to the knowledge base to improve implementation of future reablement programmes.

## Additional File

The additional file for this article can be found as follows:

10.5334/ijic.9005.s1 Appendices. Appendix 1 to 5.
