# Supplementary material for: Feasibility of a Reablement Programme in Community Care in the Netherlands; A Qualitative Study
Source: Int J Integr Care. 2025 Sep 10;25(3):27. doi: 10.5334/ijic.9005 (PMC12427612; doi:10.5334/ijic.9005)
Supplement: Appendices. — Appendix 1 to 5. [file ijic-25-3-9005-s1.pdf]

Appendix 1. Description of the implemented reablement programme based on the phases of I-MANAGE model (22)

<table>
<tr><td colspan="3">Referral of the client to the reablement programme:
- Community care nursing
- General practitioner
- Elder care physician</td></tr>
<tr><td>Start of the programme</td><td>Phase 1: Initiation
• Provide information to client (and informal caregiver)
• First visit by the occupational therapist
o Exploratory conversation using the Positive Health questionnaire (24)</td><td rowspan="5">Interdisciplinary collaboration:
- Appointed coordinator
- Regular team meetings
- Shared electronic care file
- Coaching on the job</td></tr>
<tr><td>Week 1</td><td>Phase 2: Intake
• Home visit by the occupational therapist
o Environmental assessment if needed
o Set meaningful goals using COPM
• Informal care consultant contacts informal caregiver to assess the burden and needs
• Intakes by other care professionals if necessary</td></tr>
<tr><td>Week 2</td><td>Phase 3: Care plan
• Determine interventions and actions to reach goals
o Guided by preferences of the client and informal caregiver
o Specific attention to the client's capabilities, social network and physical environment
• The reablement plan is shared and discussed with the reablement team and recorded in an electronic care file</td></tr>
<tr><td>Week 3 - 8</td><td>Phase 4: Care delivery
• Care provided as described in the reablement plan
• Bi-weekly evaluations by the reablement team
• If needed, initiate additional support for informal caregivers</td></tr>
<tr><td>Week 8</td><td>Phase 5: Evaluation
• Continuous evaluation during home visits and team meetings leading towards adaptations of the plan if needed
• Formal evaluation after 8 weeks using COPM</td></tr>
<tr><td colspan="3">Aftercare
1. Continue the programme for a maximum of 2 weeks
2. Referral to usual care if needed</td></tr>
</table>

Abbreviations: COPM; Canadian occupational performance measure.

Appendix 2. Semi-structured interview guide clients

1. How did you first encounter the reablement programme?
    a. What was your home situation like before you started the programme?
2. Could you share your experience of the initial conversation with the occupational therapist?
    a. How did you find this discussion?
    b. Were you able to talk about everything you wanted to?
    c. Did you feel that your concerns were heard?
3. What goals did you set for yourself?
    a. How did you arrive at these goals?
    b. Why are these goals significant for you?
    c. How did you find the process of setting goals?
    d. Are there any other aspects that you consider important, and were these also discussed?
4. How did you go about working towards your goals?
    a. Who did you collaborate with on this?
        i. How was your interaction with the care providers?
5. What are your thoughts on working towards goals?
    a. In what ways were you involved in the process?
6. Were there any adjustments made to the goals or the programme along the way?
    a. If so, what prompted these changes?
    b. How did you feel about these adjustments?
7. Were you able to achieve the goals you set?
8. How did the care you received differ from what you experienced before?
    a. Did the care providers effectively tailor the care to meet your needs?
    b. How well did the care providers coordinate with each other?
9. What was your overall experience of the reablement programme?
    a. What impact has the programme had on you?
    b. What aspects worked particularly well?
    c. What challenges did you face, and what didn't go as planned?
10. What helped you in working towards and achieving your personal goals?
    a. Could you elaborate on this?
11. What obstacles did you encounter that made it difficult to work towards your personal goals?
    a. Can you provide more details about this?
12. If you could make changes, what improvements would you suggest for the programme?

Appendix 3. Semi-structured interview guide informal caregiver

1. How do you currently experience caring for your loved one?
   a. What do you enjoy about caring for [your loved one]?
   b. What challenges do you face in providing care to [your loved one]?
   c. What impact does this have on your daily life/relationship with [your loved one]/your well-being?
      i. How do you cope with this?
      ii. How do you currently experience caring for your loved one?
2. Can you tell me more about the care [your loved one] received during this period?
   a. How was this different from before?
   b. What impact did this have on you as a carer?
      i. And on your relationship with your loved one?
3. How were you supported?
   a. How was your relationship with the care team?
4. How did you experience the programme?
   a. What went well?
   b. What didn't go so well/what challenges did you encounter?
5. What has the programme meant for you/what did it bring you?
   a. What has changed for you compared to before the programme?
6. What do you think enabled this programme to be carried out?
7. What do you think hindered the application of this programme?
8. Looking back on the past period, what do you think could be improved in the programme?

Appendix 4. Semi-structured interview guide professionals

1. Which factors did you experience as facilitating when implementing reablement?
    a. Why was this a facilitating factor?
2. Which factors did you experience as hindering when implementing reablement?
    a. Why was this a hindering factor?
3. What challenges did you face during the implementation of reablement?
4. How can these problems be solved?
5. What do you think are the advantages and disadvantages of reablement compared to conventional home care?
6. To what extend has current legislation and regulations influenced the implementation of reablement?
7. To what extend have other factors outside the organization influenced the implementation of reablement?
8. To what extend has the organization facilitated or hindered the implementation of reablement?
9. In what way has communication within the organization and within the reablement team influenced the implementation of reablement?
10. To what extend does reablement fit within the organization's current policy and how did this affect the implementation of reablement?
11. Which people supported you in the implementation of reablement?
    a. How did they support you?
12. Which people hindered you in the implementation of reablement?
    a. How did they hinder you?
13. Was the reablement program implemented as intended?
    a. If not, why not?
14. What strategies were used to implement the reablement program as planned?

Appendix 5. Code tree and matrix following the feasibility concepts of Bowen (2009)

Area of focus	Coordinating staff	Care professionals	Clients	Informal caregivers
**Acceptability**	- Prerequisites for implementation	- Prerequisites for implementation<br>- Broader perspective encouraging self-management and autonomy<br>- Mindset shifts	- Experiences with programme and team approach<br>- Communication with the reablement team<br>- Broader perspective encouraging self-management and autonomy<br>- Mindset shifts	- Experiences with programme and team approach<br>- Communication with reablement team
**Demand**	-	- Not everything is reablement	-	-
**Implementation**	- Building support for the programme<br>- Programme execution<br>o Supportive processes	- Building support for the programme<br>- Training<br>- Programme execution<br>o Work processes<br>o Expertise<br>o Staff availability<br>o Administration<br>o Interventions<br>o Team composition<br>o Setting goals	- Programme execution<br>o Application<br>o Work processes<br>o Domain goals<br>o Interventions used<br>o Involved professionals	- Programme execution<br>o Communication<br>o Work processes
**Practicality**	- Influence on team<br>- Funding	- Influence on team<br>- Barriers<br>- Facilitators	- Barriers<br>- Facilitators	- Barriers<br>- Facilitators
**Adaptation**		- Eligibility criteria		

**Integration**	- Support for the programme<br>- Nothing "new"	- Planning<br>- Collaboration<br>- Electronic care file<br>- Process optimisation	- Collaboration<br>- Reablement vs usual care	- Collaboration<br>- Reablement vs usual care
**Expansion**	- Policy support<br>- Scaling barriers			
**Limited efficacy testing**	- Long-term follow up	- Client satisfaction<br>- Sustaining outcomes<br>- Effects	- End result	- End result
